# Simultaneous two-color absorption dynamics in the van der Waals ferromagnet Fe_3_GeTe_2_

**DOI:** 10.1063/4.0001216

**Published:** 2026-08-04

**Authors:** Nele Stetzuhn, Emmanuelle Jal, Juliette Dubois, Boris Vodungbo, Flavio Capotondi, Emanuele Pedersoli, Matteo Pancaldi, Luca Gianessi, Claudio Masciovecchio, Ivaylo Nikolov, Giovanni Perosa, Dario De Angelis, Marco Malvestuto, Iuliia Bykova, Benedikt Rösner, Christian David, Jan Lüning, Angelo Giglia, Nicola Mahne, Deepika Gill, Sangeeta Sharma, Kirill I. Bolotin, Clemens von Korff Schmising, Stefan Eisebitt

**Affiliations:** 1 Department of Physics, Freie Universität Berlin, Arnimallee 14, 14195 Berlin, Germany; 2 Max Born Institute for Nonlinear Optics and Short-Pulse Spectroscopy, Max-Born-Str. 2A, 12489 Berlin, Germany; 3 Sorbonne Université, CNRS, Laboratoire de Chimie Physique - Matière et Rayonnement, LCPMR, F-75005 Paris, France; 4 Elettra-Sincrotrone Trieste, Strada Statale 14 km 163.5 in Area Science Park, 34149 Basovizza, Trieste, Italy; 5 Center for Photon Science, Paul Scherrer Institute, 5232 Villigen, Switzerland; 6 Helmholtz-Zentrum Berlin für Materialien und Energie, Albert-Einstein-Strasse 15, 12489 Berlin, Germany; 7 Institut für Physik, Humboldt-Universität zu Berlin, Newtonstraße 15, 12489 Berlin, Germany; 8 CNR-IOM, Strada Statale 14 km 163.5 in AREA Science Park, 34149 Basovizza, Trieste, Italy; 9 Institute of Physics and Astronomy, Technische Universität Berlin, Straße des 17. Juni 135, 10623 Berlin, Germany

## Abstract

For the integration of two-dimensional materials in future devices, a fundamental understanding of their response to external stimuli is needed. Toward this goal, we have investigated the electron and spin dynamics in the metallic van der Waals material Fe_3_GeTe_2_ (FGT) in its paramagnetic state after ultrafast optical excitation. To this end, we have employed a zone plate streaking technique with probing energies in the extreme ultraviolet range, tuned to the Fe M_2,3_ and Te N_4,5_ absorption edges. This approach provides insights into energy-dependent charge dynamics with a sensitivity to transient absorption changes on the order of $∼10^{-4}$. We find a slow carrier relaxation time at both elemental edges—up to (2.2±0.6) ps in Te and exceeding several picoseconds in Fe—which is surprising for a metal. To elucidate the complex time-resolved response, we also employ static x-ray absorption spectroscopy at the corresponding elemental edges, in which we find a double feature at the Fe M_2,3_ edge. We attribute this to different Fe sites in the pristine material and an oxidized surface layer, and we propose that the time-resolved absorption dynamics show a mixture of signals stemming from the different species. Additionally, we conducted time-resolved x-ray magnetic circular dichroism measurements in FGT at room temperature. We do not find clear evidence of the previously observed light-induced ferromagnetic order above $T_{C}$. Our study lays the groundwork for a deeper understanding of charge and spin dynamics in FGT after optical excitation as part of a roadmap for 2D spintronics.

## I. INTRODUCTION

In recent years, two-dimensional ferromagnets have attracted attention for their potential use in spintronic devices and information processing.^1,2^ Thin van der Waals magnets offer the distinct advantage of atomically flat surfaces without dangling bonds,^3–5^ allowing their combination into heterostructures with other 2D materials.^6–10^ Additionally, their reduced dimensionality leads to a strong response to external perturbations such as strain and electric fields.^11–14^ The metallic ferromagnet Fe_3_GeTe_2_ (FGT) stands out due to its comparatively high Curie temperature of ~220 K in bulk and 130 K in the monolayer,^5^ which can be increased up to room temperature by ionic liquid gating^15^ or varying concentrations of Fe and other dopants.^16,17^ Its magnetic properties are also influenced by, e.g., substrates^18^ and doping levels.^16^ Theoretically, strong electric fields^19^ and strain^11^ have further been suggested as ways to tune the ferromagnetic ordering in FGT.

Much scientific interest in ferromagnets originates from their response to short light pulses, such as ultrafast demagnetization^20^ or all-optical switching.^21–23^ FGT exhibits a two-step demagnetization process with a fast time constant of 800 fs and a slower response of several picoseconds at 80 K.^24^ The exact element-resolved response in FGT to an ultrafast excitation remains elusive, but could help understand several phenomena in the material. For example, a theoretical prediction states that immediately after the pulse, spin is transferred from the Fe to Te atoms within tens of femtoseconds.^25^ Moreover, it has been found that femtosecond laser light can transiently induce ferromagnetic order in FGT at room temperature.^26^ This has been explained by a shift of the Fermi level after photoexcitation of 3d electrons in the system. The resulting enhanced density of states at the Fermi level strengthens the magnetic order as per the Stoner theorem.^26,27^ A photoinduced increase in the Curie temperature has also been reported for the ferromagnetic semiconductor CrGeTe_3_.^28^

Time-resolved extreme ultraviolet (XUV) absorption and magnetic dichroism measurements are ideal tools for further investigation of such effects owing to ultrafast time-resolution and elemental sensitivity.^29–31^ However, due to the generally weak signal strength in such experiments, the shot-to-shot noise between consecutive pulses typical for XUV sources such as free electron lasers (FELs) poses a significant experimental challenge.^32,33^ This difficulty can be overcome by using a technique called zone plate streaking, in which an incoming XUV probe pulse is diffracted and temporally stretched by an off-axis zone plate.^34^ In the focus of the zone plate, all arrival times overlap in space, whereas, in the far field, they become spatially separated and can be recorded by a 2D detector. In this way, time-resolved information can be recorded over an extended window of several picoseconds with a single, femtosecond short pulse. Measuring multiple diffraction orders is an effective way to normalize such data, avoiding artifacts due to inhomogeneous illumination or defects in the zone plate. This technique has been further developed to allow simultaneous measurements at two different photon energies.^35^

In this work, we use the XUV zone plate streaking technique to gain information on the element-resolved response of Fe and Te in FGT to an ultrafast laser excitation at room temperature. We find an instantaneous increase in the absorption of Te when probing close to the Fermi level, followed by an energy-dependent two-step relaxation process. In Fe, we observe both positive and negative changes in absorption when probing at the lower-energy absorption edge. The positive absorption change persists for at least several picoseconds. While this behavior is surprising for a metal, we discover evidence of multiple Fe species in static absorption spectra. This could be the result of two inequivalent Fe sites in FGT, or indicate surface oxidization of the sample, possibly leading to a partially non-metallic character. Furthermore, in x-ray magnetic circular dichroism (XMCD) measurements using the same zone-plate streaking approach with the sample under an external magnetic field, we find no clear evidence for femtosecond laser-induced ferromagnetism in FGT at room temperature, in contrast to what has previously been reported in the literature.^26^

## II. SAMPLE AND EXPERIMENTAL DETAILS

**FIG. 1.**
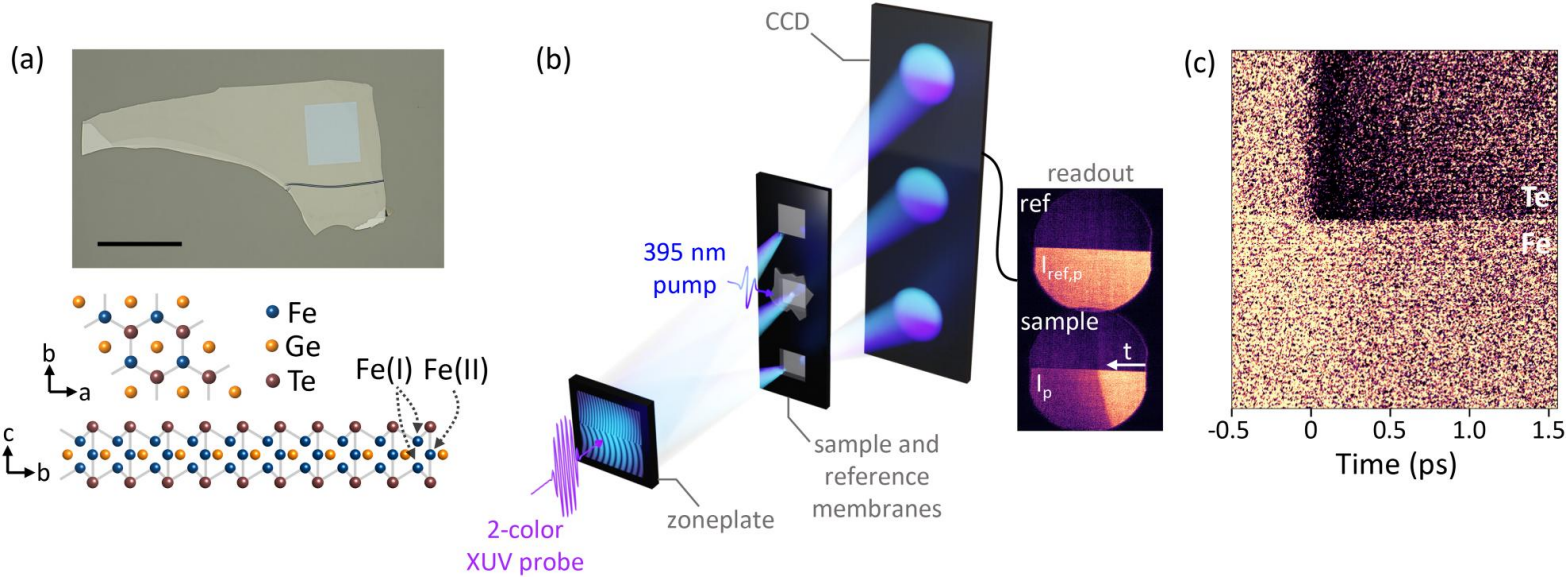
(a) Top: optical microscopy image of the FGT sample on a Si_3_N_4_ membrane used in the static absorption spectra (50 *μ*m scale bar). Bottom: top and side views of the crystal structure of FGT, showing Fe and Ge sandwiched between Te layers. There are two inequivalent sites, Fe(I) and Fe(II), in the intermediate and center layers, respectively. (b) Simplified schematic of the zone plate streaking setup. An off-axis zone plate optimized for two photon energies (with a ratio of 15:11) is used to diffract a two-color XUV probe pulse. In addition, a perpendicular grating (included in the zone plate design) creates several copies of the focus,^35^ which pass through the sample and reference membranes. The path length difference between rays of the probe diffracted at different rings of the zone plate introduces an intrinsic time delay into the probe. A pump pulse ( λ=395 nm) is synchronized to hit the sample simultaneously with part of the probe. The diverging probe beams are then imaged with a CCD behind the sample. In the recorded image, one axis contains time-resolved information (marked with *t* in the readout image). The same measurement provides a signal through the sample and a reference signal (ref) for normalization. (c) Processed image measuring absorption dynamics at the Fe $M_{2,3}$ ( E=55.02 eV, bottom half) and Te $N_{4,5}$ ( E=40.35 eV, top half) edges. The signal is then integrated along the y-axis in each half.

Like other van der Waals materials, the ferromagnetic FGT can be easily cleaved into few-layer samples along the out-of-plane direction. A single layer of FGT is ordered in a hexagonal lattice structure and consists of two outer layers of Te atoms encapsulating three layers made up of Fe and Ge. Due to their different positions in the crystal lattice in the inner vs intermediate layers, there are two inequivalent Fe sites, Fe(I) and Fe(II), in the material [Fig. 1(a)]. The FGT samples in this work were prepared by mechanical exfoliation and dry transfer in a glovebox with Ar atmosphere (0.1 ppm O_2_, 1 ppm H_2_O) to minimize sample degradation.

For the static XUV absorption measurements, we used a ∼17 nm thick FGT flake on a Si_3_N_4_ membrane [30 × 30 *μ*m^2^, 30 nm thickness, Fig. 1(a)]. The static experiments were performed at the CNR-IOM beamline at Elettra, Trieste, Italy.^36,37^ The XUV beam passed through the membrane mounted on a homebuilt sample holder with an electromagnet capable of generating an external field of up to 250 mT. The holder can be cooled down to 190 K. The transmitted intensity $I_{T}$ and the incoming intensity $I_{T,0}$ were measured with a Si photodiode in two steps (with and without the sample), while the incoming intensity was monitored using the current $I_{M}$ and $I_{M,0}$ from the last mirror of the beamline during the measurements. We then calculate the transmission of the sample as

$$T=\frac{\left(I_{T}-I_{T}^{D}\right)/\left(I_{M}-I_{M}^{D}\right)}{\left(I_{T,0}-I_{T,0}^{D}\right)/\left(I_{M,0}-I_{M,0}^{D}\right)},$$ (1)

where $I_{X}^{D}$ are the respective dark currents of the detectors. However, as the spot size is larger than the membrane—and part of the beam is subsequently cut—we cannot make quantitative statements about the absorption coefficient β∝−ln T and, in the following, show it in arbitrary units only.

To perform the XUV streaking experiments, an FGT flake ( 10–12 nm) was transferred onto a custom-made chip with a 3 × 4 array of Si(001) membranes of 100 nm thickness with a pitch of 3 mm. The sample covered most of a 50 × 50 *μ*m^2^ Si window, while two neighboring 500 × 1000 *μ*m^2^ membranes were left empty and used to pass other diffraction orders for normalization. We note here that the samples used in the dynamic and static measurements are not the same. Although they are comparable in thickness and composition (exfoliated from the same crystal), they likely differ, for example, with respect to the degree of oxidation, strain, and residues due to the fabrication process.

The working principle of the streaking technique employed at the DiProl beamline of Fermi, Trieste, Italy, has been detailed previously^34,35,38^ and is shown in a simplified manner in Fig. 1(b). The wavelength of the seed laser of the FEL is tuned so that the 15th and 11th harmonics generated in the undulator of the FEL match the Fe $M_{2,3}$ and Te $N_{4,5}$ absorption edges, respectively. The resulting two-color XUV pulse (pulse energy 4 *μ*J, repetition rate 50 Hz) is then diffracted at the zone plate. The off-axis zone plate consists of two halves (2.5 × 5 mm^2^ each) with different grating pitches designed to focus each wavelength in the same focal plane ( 75 mm), respectively. The zone plate is manufactured on a 200 nm thick Si membrane, etched down to 120 nm within the window areas. Details on the manufacturing process can be found elsewhere.^35^ In the sample plane, there are multiple focused beams (spot size ∼10 × 30 *μ*m^2^) belonging to the zeroth and ± first diffraction order of the zone plate. While one of them passes through the FGT sample, the others can be used as reference beams transmitted through empty membranes. After the sample plane, the beams diverge and produce images of the zone plate on the CCD camera. The images contain the time-resolved information of the probe along one axis, as the light diffracted at different rings of the zone plate has a different path length to the sample depending on the ring's radius. Effectively, the zone plate therefore introduces a time delay between parts of the same pulse.

We then introduce an optical pump laser ( $\lambda_{\text{pump}}=395\text{ nm}$, $\tau_{\text{pulse}}=90\text{ fs}$, fluence of $9.8–11.8\text{mJ}{\text{cm}}^{-2}$) and synchronize it such that it arrives on the sample during the time window probed by the streaked XUV pulse. Therefore, one shot of the XUV probe contains information about the system before, during, and after excitation. The CCD then records the reference signal $I_{\text{ref},p}$ as well as the signal through the pumped sample $I_{p}$. Even in the non-normalized image, a clear change in intensity after time delay zero $t_{0}$ is visible [Fig. 1(b)]. We normalize $I_{p}$ by $I_{\text{ref},p}$ for each pulse and average over multiple pulses. Additionally, we record images without optical pump, yielding $I_{\text{up}}$ and $I_{\text{ref},\text{up}}$, which we use to calculate the absolute value of the change in transmission. As a last step, we correct for the curvature caused by the zone plate rings, as well as an additional tilt introduced by the perpendicular grating in the ± 1st diffraction order.^35^ The resulting transmitted intensity [Fig. 1(c)] is then

$$T=\frac{I_{p}/I_{\text{ref},p}}{I_{\text{up}}/I_{\text{ref},\text{up}}},$$ (2)

from which we calculate the change in absorption in relative units as ΔXAS=−ln(T).

## III. STATIC AND TRANSIENT X-RAY ABSORPTION SPECTROSCOPY (XAS) MEASUREMENTS AT THE Fe M_2,3_ EDGE OF FGT

**FIG. 2.**
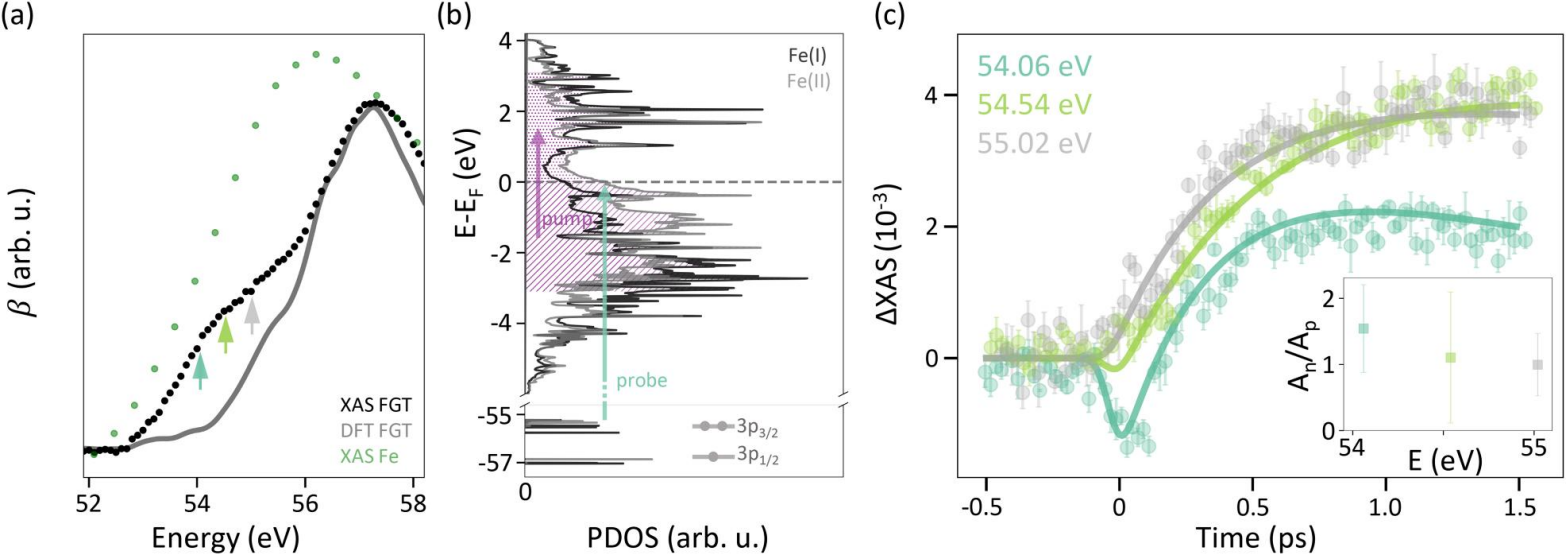
(a) Static spectrum of the absorption coefficient β in arbitrary units at the Fe $M_{2,3}$ edge. The absorption measured on FGT (black data points) shows a double feature at higher energies compared to data measured on pure Fe (green data points, from Ref. 39). The splitting in the FGT spectrum calculated with DFT (gray solid line) is smaller than observed in the experiment. (b) Partial density of states of the Fe(I) (black) and Fe(II) (gray) 3d- and 3p-orbitals. The UV pump pulse (purple arrow) excites d-electrons above the Fermi energy in the streaking experiments (dotted), leaving behind empty states (striped) for the probe (blue arrow). We probe transitions from the $3p_{1/2}$ and $3p_{3/2}$ levels into 3d orbitals. Due to the energy difference of the p-orbitals of Fe(I) and Fe(II), we probe at different energies with respect to $E_{F}$ for the two species. (c) Measured transient absorption changes at 54.06 eV (dark green), 54.54 eV (bright green), and 55.02 eV (gray). The corresponding energies are marked with arrows of the same color in (a). Measurement data are shown as points and fits as solid lines. The inset shows the ratio of the fitted amplitudes $A_{n}$ and $A_{p}$ of the negative and positive changes in absorption, respectively.

In the static XUV absorption measured around the Fe $M_{2,3}$ edge in FGT, we observe a double feature with a shoulder around ∼54.5 eV and a peak at ∼57.3 eV [black data points in Fig. 2(a)]. The absorption at these energies is the result of transitions from Fe 3p to 3d orbitals. Compared to a spectrum measured on pure Fe [green data points in Fig. 2(a), taken from Ref. 39], the absorption edge in FGT is blueshifted to higher photon energies by ∼1–2 eV. Furthermore, there is only a single feature in the pure Fe data. The calculated XAS spectrum for FGT, obtained by density functional theory (DFT) [gray line in Fig. 2(a)], reproduces the shoulder at lower energies, even though the splitting between the two features in the theoretical spectrum is smaller than that observed in the experiment.

We hypothesize that the spectral shape at the Fe $M_{2,3}$ edge in FGT originates from multiple oxidation states ${\text{Fe}}^{2+}$ and ${\text{Fe}}^{3+}$ present in the sample, as the absorption at these energies is sensitive to their presence. Generally, the existence of ${\text{Fe}}^{2+}$ and ${\text{Fe}}^{3+}$ results in a double feature in absorption, which is higher in energy than in ${\text{Fe}}^{0}$.^40–42^ The relative intensities and peak splitting of this feature depend on the relative concentrations of the two species.^41^ Different Fe states exist in FGT naturally due to the two inequivalent sites Fe(I) and Fe(II), explicitly $\left({\text{Te}}^{2-}\right)\left(\text{Fe}{\left(I\right)}^{3+}\right)[\left(\text{Fe}{\left(\text{II}\right)}^{2+}\right)\left({\text{Ge}}^{4-}\right)]\left(\text{Fe}{\left(I\right)}^{3+}\right)\left({\text{Te}}^{2-}\right)$.^15,43,44^ Consistent with this, we observe a core-level shift resulting from the different environments of the Fe sites in the density of states (DOS) calculated with DFT [gray and black lines in Fig. 2(b)], visible in the structure of the theoretical absorption edge in Fig. 2(a). The remaining discrepancies between the theoretical and measured static spectra in Fig. 2(a) could have several underlying reasons. On the one hand, it has been reported that FGT crystals host randomly distributed Fe atoms in the van der Waals gap.^45^ This and other lattice defects can influence the shape of the absorption edge, as their crystallographic surroundings differ from those of Fe(I) and Fe(II). Additionally, the formation of an oxidized surface layer (O-FGT) can result in further changes to the sample composition. During the surface oxidization, the Fe in FGT first turns into Fe^2+^O, then ${\text{Fe}}_{2}^{3+}$O_3_, and finally ${\text{Fe}}^{2+}$CO_3_.^46^ Although the samples were prepared in a protected atmosphere, they were exposed to the environment during transportation and mounting (max. 1 h for the sample used in static and max. 24 h for the sample used in time-resolved measurements). Previous reports^46–49^ have described the surface oxidization of Fe_3_GeTe_2_ within minutes, followed by the complete oxidation of the sample (the duration of which depends on the thickness of the sample). We conclude that our samples can have an oxide layer of up to a few nm.

**FIG. 3.**
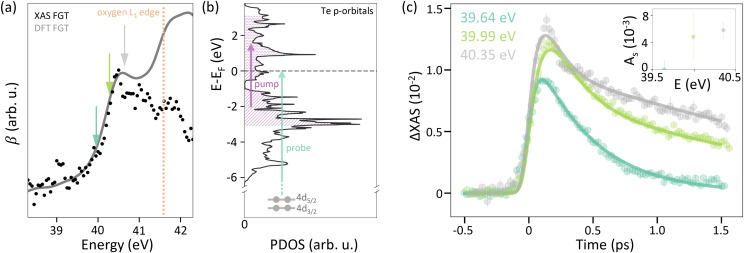
(a) Static spectrum of the absorption coefficient β in arbitrary units at the Te $N_{4,5}$ edge measured on FGT (black data points) and calculated with DFT (gray solid line). The theoretical spectrum shows two features at the edge due to transitions from the $4d_{3/2}$ and $4d_{5/2}$ core levels, respectively. In the experimental spectrum, only one feature is clearly visible. The theoretical position of the oxygen $L_{1}$ edge is shown as the orange dashed line. (b) Partial density of states of the Te p-orbitals around the Fermi energy. The UV pump pulse (purple) excites electrons above the Fermi energy (dotted) in the streaking experiments, leaving behind empty states below $E_{F}$ (striped). We probe excitations from the $4d_{3/2}$ and $4d_{5/2}$ levels into 4p orbitals (blue arrow). (c) Differential absorption ΔXAS at 39.64 eV (dark green), 39.99 eV (bright green), and 40.35 eV (gray). The corresponding energies are marked with arrows of the same color in (a). The measurements (dots) were conducted simultaneously with those in Fig. 2(c) with the same color. The solid lines are fits to the data. The inset shows the fitted amplitude $A_{s}$ of the slow decay component, which increases with energy.

In Fig. 2(c), we show the ΔXAS curves measured in the streaking experiments using probe energies of 54.06 eV (dark green, averaged from 2 images with 150 shots each), 54.54 eV (light green, averaged from 3 images with 35 shots, 1 image with 200 shots), and 55.02 eV (gray, 1 image with 120 shots). The time of pump-probe overlap $t_{0}$ was determined by fitting the data at the Te edge [Fig. 3(c)]. Several details of the behavior of the transient ΔXAS after excitation are surprising. At the lowest probe energy, a decrease in absorption is visible directly after the arrival of the pump. With increasing probe energy, this component is weakened, its effect only visible in the rise time of the positive signal component. This positive change in absorption exhibits a remarkably slow recovery exceeding our probed time window of 1.6 ps (set by the design of the off-axis zone plate).

To quantify these trends, we choose the fit function,

$$\Delta \text{XAS}=A_{p}\cdot \text{EMG}(\tau_{d,p},\sigma )-A_{n}\cdot \text{EMG}(\tau_{d,n},\sigma ),$$ (3)

where EMG are exponential functions convolved with a Gaussian,

$$\text{EMG}\left(\tau ,\sigma \right)=e^{\frac{\sigma^{2}}{2\tau^{2}}-\frac{t}{\tau }}\left(1-\text{erf}\left(\frac{\sigma^{2}/\tau -t}{\sqrt{2}\sigma }\right)\right).$$ (4)

The width of the Gaussian σ is fixed by the duration of the pump pulse of ∼90 fs. The first term in Eq. (3) describes the positive change in absorption with amplitude $A_{p}$ and decay time $\tau_{d,p}$. The negative component is equivalently determined by the amplitude $A_{n}$ and decay time $\tau_{d,n}$. Equation (3) describes the behavior in Fig. 2(c) sufficiently without the inclusion of explicit rise times, suggesting that they are shorter or on the order of the pulse length. The fitting parameters are summarized in Table I.

**TABLE I.** Fitting results for the transient absorption traces at the Fe edge in FGT. This table summarizes the fits of the time traces in Fig. 2(c) to Eq. (3) for different probe energies *E*. $A_{n}$ and $A_{p}$ are the amplitudes of the negative and positive components, respectively. The decay times of the two components are $\tau_{d,n}$ and $\tau_{d,p}$. For the higher energies, the decay of the positive component is too slow for a meaningful fit with the available data points. The errors are estimated from the covariance matrix of the least squares fits.

*E* (eV)	$A_{n}(10^{-3})$	$A_{p}(10^{-3})$	$\tau_{d,n}$ (ps)	$\tau_{d,p}$ (ps)
54.06	2.9±0.5	1.9±0.5	0.35±0.07	2.5±1.0
54.54	3.0±1.0	2.7±1.3	0.6±0.2	>Probe window
55.02	2.2±0.5	2.2±0.6	0.4±0.1	>Probe window

Comparing the relative intensities of the positive and negative contributions across energies, we find that for the lowest energy $A_{n}/A_{p}>1$, resulting in the aforementioned initial decrease in ΔXAS. For the higher probe energies, $A_{n}$ and $A_{p}$ become comparable [see the inset of Fig. 2(c)]. For all probe energies, the negative component decays quickly, with time constants between $\tau_{d,n}=(350\pm 70)\text{ fs}$ and $\tau_{d,n}=(600\pm 200)\text{ fs}$. Conversely, the positive component is long-lived, with a minimum value of $\tau_{d,p}=(2.5\pm 1.0)\text{ ps}$ at 54.06 eV, and even exceeding the probed window for the two higher energies.

To understand the observed energy-dependent dynamics, it is necessary to understand which transitions make up the time-resolved signal. The UV pump pulse excites electrons to states above the Fermi level [purple arrow in Fig. 2(b)], leading to a temporary redistribution of electrons around $E_{F}$. This allows transitions into states that were previously occupied. We mark the states that can be excited by the pump pulse with the striped area in the partial density of states in Fig. 2(b), and the possible states after excitation with dots. Probing states below $E_{F}$ (striped) will result in an increased absorption after $t_{0}$, probing states above (dotted) will result in a decrease. In that sense, the ΔXAS traces in Fig. 2(c) suggest that we probe states above the Fermi energy at all probe energies. Comparing the range of probe energies in the streaking experiments (between 54 and 55 eV) to the static XAS spectrum, they lie along the maximum of the low-energy absorption feature [arrows in Fig. 2(a)]. Provided that the observed splitting is a result of different iron species in FGT, this would mean that we are probing the dynamics of the two species at different transitions. Explicitly, we probe transitions very close to the Fermi edge of Fe(II), and transitions below $E_{F}$ for Fe(I). This could result in overlapping transient components with different dynamics and amplitudes in Fig. 2(c). We note that the relative intensities of the features in the XAS spectrum of the sample measured in the time-resolved measurements could be slightly different from the ones shown in Fig. 2(a) due to the different sample thicknesses and, accordingly, the different relative degree of oxidation. However, we estimate that within the exposure times of max. 1 h for the sample measured at Elettra and max. 24 h for the sample measured at Fermi, the absolute thickness of the oxidation layer will only be a few nanometers in both cases.^46,50^

The energy dependence of the negative transient absorption change $A_{n}$ observed at the Fe $M_{2,3}$ edge is particularly puzzling. Several effects taking place after the pump pulse, such as heating and electronic state blocking, can lead to a dynamic reshaping of the XAS spectrum directly after excitation, resulting in a shift of the absorption edge to lower energies.^29,42,51–55^ This results in an increase in the time-resolved absorption spectrum below $E_{F}$ and a decrease above, which can lead to two time-resolved components with opposite signs when probing close to the Fermi level. However, this effect should be stronger the closer the probe energy is to the Fermi energy. We would therefore expect an increase in $A_{n}$ with increasing probe energy. In our experiments, we see the exact opposite trend [inset of Fig. 2(c)]: The negative component is the most pronounced when probing furthest from the first peak in the static XAS spectrum [dark green arrow in Fig. 2(a)] and becomes weaker when crossing the resonance. Therefore, we conclude that electron redistribution or heating effects cannot explain our experimental observations. There are alternative phenomena that could be the underlying reason for the energy-dependent negative component in Fig. 2(c). For example, it is possible that the oxidation state of either Fe site in FGT transiently changes due to charge transfer between the different elements or species within the sample.^56,57^ The change in screening could modify the density of states at the Fermi level, as well as the shape of the absorption spectrum in Fig. 2(a). However, the complexity of the sample composition as well as the small number of energy-dependent time traces ultimately does not allow us to draw a final conclusion.

The dynamics of the positive component of ΔXAS in Fig. 2(c) can mostly be explained by heating of the sample after pump excitation. In metals, the creation of non-equilibrium electrons above $E_{F}$ is usually assumed to be instantaneous with the pump pulse, followed by a relaxation back to a Fermi distribution within hundreds of femtoseconds.^58–62^ This relaxation is slower when probing at the Fermi level (as the smearing of the distribution around $E_{F}$ scales with the electron temperature), which could explain the increase in the slow recovery component $\tau_{d,p}$ in Table I. However, our measured decay times exceed the typical thermalization timescales by several ps. This could have several underlying mechanisms.

On the one hand, the two different Fe sites in the material may exhibit different dynamics. FGT has been described as a site-differentiated Hund metal,^44^ in which the electronic structure—and, in particular, the itinerant character of the electrons—at the Fe(I) and Fe(II) sites differs. One of the sites could therefore have a longer thermalization timescale. However, we note that stronger correlation effects are expected for Fe(I),^44^ which we tentatively assigned to the higher-energy feature in Fig. 2(a). Second, we cannot exclude that the dynamic processes in Fig. 2(c) are affected by the complexity in sample composition due to oxidization. As oxidized Fe is no longer metallic, additional phenomena such as electron trapping at oxidized sites, relaxation into mid-gap states, or electron–hole recombination can occur. In other oxidized Fe compounds such as γ- or α-Fe_2_O_3_, these processes result in charge dynamics exceeding tens of picoseconds.^63,64^ We thus might measure a mixture of contributions from both FGT as well as O-FGT, which makes a clear assignment of the exact physical processes behind the dynamics challenging. In particular, the amplitudes of the different dynamical components could differ depending on the degree of oxidation if they stem from the O-FGT layer. For future XUV experiments on FGT, it is important to avoid oxidation, which could, for example, be achieved by depositing a protective Ge capping layer.^46^ Additionally, a measurement with a broadband XUV pulse would be advantageous to disentangle multiple effects in the dynamics.^54^

## IV. STATIC AND TRANSIENT XAS MEASUREMENTS AT THE Te $N_{4,5}$ EDGE OF FGT

In the second part of this work, we discuss the static and dynamic absorption of the Te atoms in FGT. In the static absorption spectrum in Fig. 3(a), we observe a prominent feature around 40.5 eV, which we attribute to the Te $N_{4,5}$ edge (black data points). As this edge consists of two transitions from the ${4d}_{5/2}$ and ${4d}_{3/2}$ into 4p orbitals, we expect two spectral features as predicted in the DFT XAS spectrum [gray solid line in Fig. 3(a)]. There indeed is a second, weak feature around 41.5 eV in the experimental data. However, we cannot clearly assign it to the Te edge: Just as Fe, the Te in FGT is subject to oxidation, which can lead to changes in the static absorption spectrum.^46^ The presence of oxygen can also result in a feature at the oxygen $L_{1}$ edge at 41.6 eV [orange dashed line in Fig. 3(a)], which partially overlaps with the second feature expected from the DFT calculations of the Te $N_{4,5}$ edge. The low photon count in this energy range makes it difficult to resolve this complex feature.

The zone plate streaking technique allows us to measure dynamics at two selected energies simultaneously. The energy pairs measured at the same time are marked with the same colors in Figs. 2(a) and 2(c) and Figs. 3(a) and 3(c). The probe energies of 39.64, 39.99, and 40.35 eV are chosen along the prominent low-energy feature [arrows in Fig. 3(a)]. In the partial density of states of Te in Fig. 3(b), the two lower probe energies correspond to transitions just below $E_{F}$, while 40.35 eV is directly at the Fermi energy. In the streaked time traces, we again observe an increase in absorption after $t_{0}$ for all probe energies in Fig. 3(c), as the pump pulse [purple arrow in Fig. 3(b)] frees up previously occupied states [striped area in Fig. 3(b)]. We find energy-dependent, two-component recovery dynamics in the dynamic data at the Te edge. To accurately describe the onset of the time traces, we also need to include the rise time dynamics in the fits. We therefore choose the function

$$\begin{array}{cc} \Delta \text{XAS}=A_{f}(\text{EMG}(\tau_{d,f},\sigma )-\text{EMG}(\tau_{r,f},\sigma )) & \\ +A_{s}(\text{EMG}(\tau_{d,s},\sigma )-\text{EMG}(\tau_{r,s},\sigma )). & \end{array}$$ (5)

The fast component is described by an amplitude $A_{f}$ as well as a rise time $\tau_{r,f}$ and decay time $\tau_{d,f}$. Similarly, the slow counterpart is captured by amplitude $A_{s}$ and the time constants $\tau_{r,s}$ and $\tau_{d,s}$. The fitting parameters are summarized in Table II. Due to the less ambiguous dynamics around $t_{0}$ in the traces in Fig. 3(c) compared to Fig. 2(c), we fit the exact value of $t_{0}$ with the traces at the Te edge and fix it to the same value for all other fits. We see no delay in the onset of the dynamics at the Fe and Te edges.

**TABLE II.** Fitting results for the transient absorption traces at the Te edge in FGT. This table summarizes the fits of the time traces in Fig. 3(c) to Eq. (5) for different probe energies *E*. $A_{f}$ and $A_{a}$ are the amplitudes of the fast and slow positive components, respectively. The rise times of the two components are $\tau_{r,f}$ and $\tau_{r,s}$, and the decay times are $\tau_{d,f}$ and $\tau_{d,s}$. The errors are estimated from the covariance matrix of the least squares fits.

*E* (eV)	$A_{f}(10^{-3})$	$A_{s}(10^{-3})$	$\tau_{r,f}$ (ps)	$\tau_{r,s}$ (ps)	$\tau_{d,f}$ (ps)	$\tau_{d,s}$ (ps)
39.64	6.9±0.5	∼0	≤0.09	⋯	0.4±0.2	⋯
39.99	20±30	4.8±3.3	120±40	0.3±0.6	0.2±0.2	1.7±1.0
40.35	20±30	5.8±1.1	≤0.09	0.2±0.2	0.1±0.1	2.2±0.6

We make several observations in the dynamics at the Te $N_{4,5}$ edge: First, the rise time of the fast component, $\tau_{r,f}$, is nearly energy independent. Second, the fast recovery component speeds up from $\tau_{d,f}=(400\pm 200)\text{ fs}$ for 39.64 eV to $\tau_{d,f}∼100\text{ fs}$ for 40.35 eV. The slow component, described by the amplitude $A_{s}$, is negligibly small at the lowest energy and increases as the probe energies approach the Fermi energy [see inset of Fig. 3(a)].

Concurrently, the time constant of the slow decay $\tau_{d,s}$ increases from $\tau_{d,s}=(1.7\pm 1.0)\text{ ps}$ at 39.99 eV to $\tau_{d,s}=(2.2\pm 0.6)\text{ ps}$ at 40.35 eV.

The underlying mechanisms of the energy-dependent dynamics at the Te $N_{4,5}$ edge are manifold. The fast increase within $\tau_{r}$ can be attributed to the generation of hot electrons leaving behind hole states below the Fermi energy, allowing new transitions for the XUV probe pulse. When the hot electrons scatter and relax, the absorption decreases again within $\tau_{d,f}$. These time constants in our experiments are similar to the characteristic heating and cooling times of $\tau_{r}=(15\pm 5)\text{ fs}$ and $\tau_{d}=(380\pm 90)\text{ fs}$ measured at the Te $N_{4,5}$ edge in MoTe_2_^65^ or $\tau_{d}∼300\text{ fs}$ in elemental Te.^66^

There can be multiple origins of the subsequent slow decay within $\tau_{d,s}$. Heating the sample with the pump pulse leads to a broadening of the Fermi–Dirac distribution of electrons at the Fermi level. As the long-lived component becomes prominent when probing close to $E_{F}$, we suggest that there is a residual temperature of the electrons remaining after the initial cooling within $\tau_{d,f}$. In the semiconducting elemental Te, multiple carrier relaxation times between 0.3 ps and >100 ps have been reported and attributed to stepwise cooling of carriers between subbands.^66^ In metallic FGT, we, in principle, do not expect such effects. However, as discussed in Sec. III on the dynamics at the Fe edge, we cannot exclude effects of oxidation of Te resulting in a partially non-metallic character of the sample.

## V. XMCD IN FGT AT ROOM TEMPERATURE

Finally, we briefly discuss time-resolved x-ray magnetic circular dichroism (XMCD) measurements on FGT at room temperature. It has been suggested that an increased electron density at the Fermi energy after femtosecond laser excitation may induce ferromagnetism in FGT above its Curie temperature of ∼220 K.^26^ The resulting spin-dependent density of states should lead to a difference in absorption for circularly polarized light in opposite magnetic fields. To this end, we install a movable pair of permanent magnets close to the FGT sample and compare the absorption of circularly polarized light for opposite fields [the coercive field is expected to be <100 mT (Ref. 26)]. The pump laser in our experiments ( $\lambda_{\text{pump}}=395\text{ nm}$, $\tau_{\text{pulse}}=90\text{ fs}$, and fluence of $9.8–11.8\text{mJ}{\text{cm}}^{-2}$) has similar parameters to those used in Ref. 26 ( $\lambda_{\text{pump}}=400\text{ nm}$, $\tau_{\text{pulse}}=50\text{ fs}$, and energy density of $∼0.6–5\text{mJ}{\text{cm}}^{-2}$). While the energy density in our experiments was higher and our sample thicker, the photoinduced ferromagnetism reportedly increased with both fluence and layer number.^26^

**FIG. 4.**
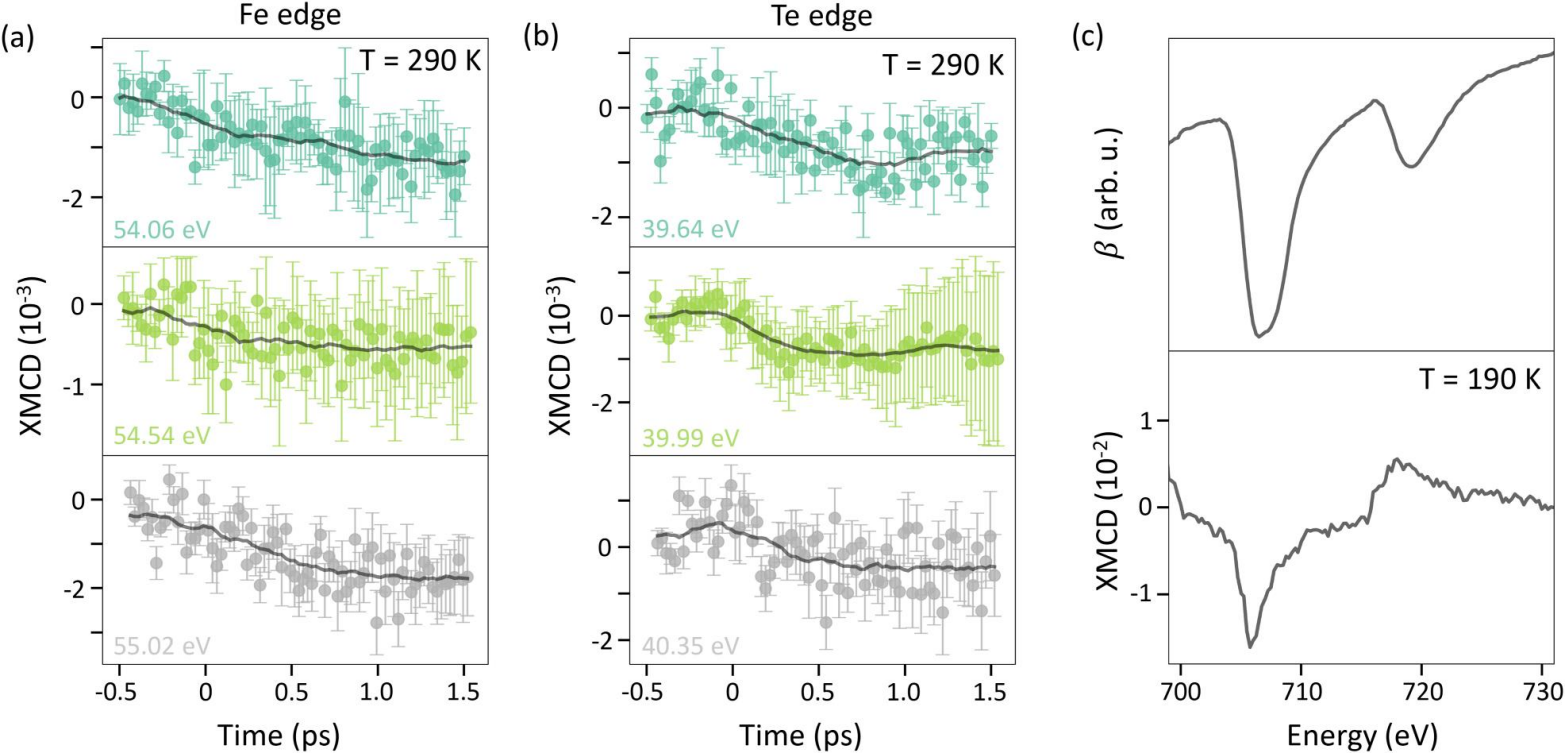
Time-resolved XMCD measurements at the (a) Fe $M_{2,3}$ and (b) Te $N_{4,5}$ edges at room temperature. The rolling mean of the XMCD is indicated by the black line as a guide to the eye. (c) Static absorption and XMCD at the Fe $L_{2,3}$ edges at 190 K, showing the ferromagnetic nature of the sample below Curie temperature.

We calculate the magnetic dichroism as $\text{XMCD}=\text{ln }\left(\frac{T_{+}}{T_{-}}\right)$, where + and − denote subsequent scans at opposite magnetic field directions (we assume no XMCD before $t_{0}$ and overlap the traces there accordingly). In Fig. 4, we show the resulting traces for all energy pairs, where the error bars are determined using the standard error calculated from different scans. As a guide to the eye, we show the mean XMCD in all traces (black lines in Fig. 4).

While we see small changes in XMCD across the probed time window, there are several observations which prohibit a claim of room-temperature induced ferromagnetism in this sample. First, we observe signal changes only on the order of $\leq 10^{-3}$, which is on the same order of magnitude as the error bars. This upper bound on the induced XMCD must be considered in relation to the substantial light-induced Kerr rotations reported by Liu *et al.*^26^ Previous studies at the FGT Fe L-edge at low temperatures yielded XMCD values of several percent,^67–69^ and the expected ratio of XMCD amplitudes at the 2p (L-edge) and 3p (M-edge) resonances scales as $\frac{\lambda_{3p}\Delta \beta_{2p}}{\lambda_{2p}\Delta \beta_{3p}}\approx 1.6$.^39,70^ Our observed transient XMCD magnitudes of $\leq 10^{-3}$ are therefore inconsistent with the claim of significant light-induced magnetization. Second, the changes in XMCD [in particular, in Fig. 4(a)] partially occur before the pump pulse at $t_{0}$. Third, when looking at single scans, the sign and magnitude of the pump-induced signal change are not always reproducible. This points toward the apparent changes in XMCD stemming from artifacts persisting after the normalization, for example, due to small drifts of the sample between scans when changing the magnet position.

We, therefore, conclude that we do not find convincing evidence for ultrafast-induced ferromagnetism in FGT at room temperature within our experimental precision. We note, however, that we cannot exclude that the likely presence of oxidized FGT lowers the induced magnetic signal strength. To verify that the samples remained at least partially magnetic, we measured the static XMCD at the Fe $L_{2,3}$ edges at 190 K, i.e., just below the Curie temperature (same sample as used for the static XAS data). We find a magnetic contrast of ∼1.6% at the ${2p}_{3/2}$ transition. Estimating the expected signal strength for a 17 nm thick sample using previously reported values^69^ and correcting for the difference in temperature,^50^ we arrive at ∼1.2%–1.8%. Additionally, we performed optical temperature-dependent hysteresis measurements on samples with a similar thickness and ambient exposure time and find magnetic behavior (Appendix). We therefore exclude complete sample oxidization. Notably, in the previous report of photoinduced ferromagnetism in FGT,^26^ the absence of a capping layer could also have led to an oxidized surface. Finally, we add that the samples in Ref. 26 were grown by molecular-beam epitaxy, while we use mechanical exfoliation from a commercial crystal by HQ graphene.

## VI. CONCLUSION

In summary, we have shown the first time-resolved XUV absorption measurements at the Fe $M_{2,3}$ and Te $N_{4,5}$ edges in the two-dimensional ferromagnet FGT. The applied zone plate streaking technique allows us to resolve very small changes in the absorption down to ΔXAS $∼10^{-4}$, demonstrating its high sensitivity. At both resonances, we observe complex energy-dependent dynamics when probing close to the Fermi energy. In particular, we observe prolonged relaxation times of the transient absorption changes for both elements, which contradicts the metallic nature of FGT. The structure of the static XUV absorption spectra at the Fe edge is a superposition of contributions from two different Fe sites in the structure, as well as Fe defects and an oxidized surface layer. We can therefore not give a definitive answer on whether the long-lived transient absorption is, e.g., an effect of site-differentiated Hund metallicity in FGT or a result of the insulating oxidized FGT. Additionally, we conducted room temperature XMCD measurements to investigate possible signs of light-induced ferromagnetic order. Within our experimental uncertainty of $∼10^{-3}$, we do not find evidence for an induced magnetization after a femtosecond pump excitation.

This work opens the path to future experiments aiming to elucidate the charge and spin dynamics of individual elements in FGT or its heterostructures. If the Fe $M_{2,3}$ edge in FGT indeed allows separate excitation and probing of Fe(I) and Fe(II) within the crystal structure, this would enable site-resolved measurements of absorption and magnetic contrast. The interpretation of future experiments will greatly benefit from broadband transient spectra across the Te and Fe resonances. Furthermore, our study indicates that any oxidation of the FGT surface has to be avoided.

## Figures and Tables

**FIG. 5.**
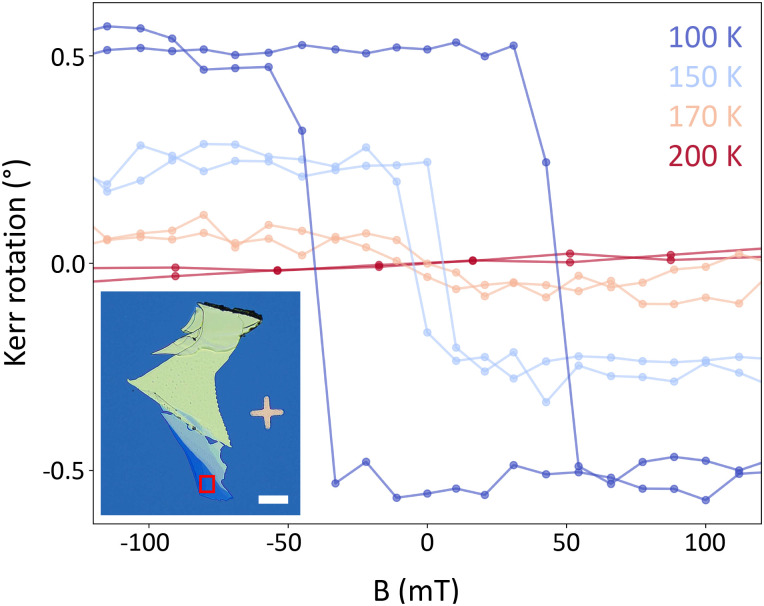
Hysteresis measurements on thin FGT as a function of temperature measured using MOKE microscopy. The inset shows an optical image of the FGT sample used; the red area indicates the measurement spot on a thin (<10 nm) part of the flake. The scale bar corresponds to 20 *μ*m.

## Data Availability

The data that support the findings of this study are openly available in Zenodo at https://doi.org/10.5281/zenodo.19555460, Ref. 71.

## APPENDIX: TEMPERATURE-DEPENDENT OPTICAL HYSTERESIS MEASUREMENTS ON THIN FGT

In order to examine the degree of oxidation of the thin FGT samples after ambient exposure, we conducted additional temperature-dependent hysteresis measurements using magneto-optical Kerr (MOKE) microscopy.^23^ Figure 5 shows the hysteresis curves of an FGT flake (<10 nm thick) after being exposed to air for about 24 h. It exhibits the typical magnetic response of magnetic FGT, with a Curie temperature of approximately 200 K. Therefore, despite the presence of an oxidized surface layer, we conclude that the samples used in this study are predominantly magnetic FGT.
